# Comparative evaluation of metformin and liraglutide cardioprotective effect in rats with impaired glucose tolerance

**DOI:** 10.1038/s41598-021-86132-2

**Published:** 2021-03-23

**Authors:** Anna Simanenkova, Sarkis Minasian, Tatiana Karonova, Timur Vlasov, Natalya Timkina, Oksana Shpilevaya, Aleksandra Khalzova, Anzhelika Shimshilashvili, Valeria Timofeeva, Daniil Samsonov, Yury Borshchev, Michael Galagudza

**Affiliations:** 1 Almazov National Medical Research Centre, Saint-Petersburg, Russia; 2 Pavlov First Saint-Petersburg State Medical University, Saint-Petersburg, Russia

**Keywords:** Diabetes, Pre-diabetes, Cardiology, Circulation

## Abstract

Impaired glucose tolerance (IGT) increases cardiovascular risk and can enlarge myocardial infarction (MI) incidence and severity. There is lack of information about cardioprotective potential of glucose-lowering drugs in IGT. We aimed to evaluate the sustainability of myocardium to ischemia–reperfusion injury in diabetic and IGT rats and to study cardioprotective action of metformin and liraglutide. Type 2 diabetes mellitus (DM) and IGT were modelled in Wistar rats by high-fat diet and streptozotocin + nicotinamide. 4 weeks after rats were divided into 4 groups: DM (without treatment) (n = 4), IGT (without treatment) (n = 4), IGT + MET (metformin 200 mg/kg per os once daily 8 weeks) (n = 4), IGT + LIRA (liraglutide 0.06 mg/kg s.c. once daily for 8 weeks) (n = 4). Control (n = 6) and high-fat diet (n = 8) groups were made for comparison. After 8 weeks ischemia–reperfusion injury in isolated hearts was performed. Hemodynamic parameters were evaluated and MI size was measured by staining of myocardium slices in triphenyltetrazolium chloride solution. Blood glucose level was measured during the study. Both IGT and DM led to similar worsening of hemodynamic parameters during ischemia–reperfusion period, in comparison with control group. MI size in IGT (56.76 (51.58; 69.07) %) and DM (57.26 (45.51; 70.08) %) groups was significantly larger than that in control group (42.98 (33.26; 61.84) %) and did not differ between each other. MI size in high-fat diet group (56.98 (47.11; 62.83) %) was as large as in IGT and DM groups (*p* > 0.05). MI size in IGT + MET (42.11 (38.08; 71.96) %) and IGT + LIRA (42.50 (31.37; 60.40) %) was smaller than in both DM and IGT groups (*p* < 0.05 for multiple comparison). Myocardium damage size did not differ in IGT + MET and IGT + LIRA groups (*p* >  0.05). Only LIRA, but not MET administration to IGT rats led to ischemic contracture reduction. Glycemic control was similarly satisfactory in IGT, IGT + MET, IGT + LIRA groups. Thus, IGT and DM have similarly pronounced negative influence on hemodynamics and MI size in rat transient global ischemia ex vivo. Obesity development also has negative impact on the MI size. Both MET and LIRA have infarct-limiting effect independent on their influence on glucose level. LIRA, but not MET, diminishes ischemic contracture in IGT rats.

## Background

Type 2 diabetes mellitus (DM) is one of the leading problems of modern health care. According to the International Diabetes Federation (IDF) Atlas, there are 463 million people with DM in the world in 2019, and their number is growing steadily^1^. In parallel with the progressive increase in the number of patients with DM, the number of people with prediabetes is rapidly increasing. Thus, in 2019, there are almost 374 million people in the world with an identified impaired glucose tolerance (IGT), which means that every thirteenth adult has this variant of impaired carbohydrate metabolism^1^.

It is well known that the main damage caused by DM is due to the development of chronic and acute disease complications^2,3^. Cardiovascular accidents, including myocardial infarction (MI), have a leading position in the structure of type 2 diabetic patients’ mortality^1,4,5^. At the same time, data on the prevalence and clinical manifestations of cardiovascular diseases, including MI, in persons with different forms of prediabetes are partly contradictory^6,7^, although most investigations demonstrate increased cardiovascular risk in prediabetes.

Thus, a meta-analysis published in 2016 by Yuli Huang et al. that included 53 prospective cohort studies with 1,611,339 individuals revealed that comparing with normoglycemia, both impaired fasting glycemia (IFG) and IGT increase the composite cardio-vascular risk for 1.13 and 1.30 times, respectively. Besides, both IFG and IGT enlarge coronary artery disease and all-cause mortality risk with IGT having more pronounced negative influence^6^.

Conversely, another meta-analysis of 20 studies showed that IGT increases the risk of cardiovascular disease by more than 1.5 times, compared with the absence of impaired glucose metabolism, while IFG does not^8^. Similarly, the Funagata Diabetes Study, a 7-year prospective study conducted in the Japanese population, showed that IGT, but not IFG, is a risk factor for cardiovascular disease^9^. The GAMI (Glucose Tolerance in Patients with acute myocardial infarction) study revealed that IGT is a common variant of glucose metabolism disorders among patients with MI^10^ and increases the risk of subsequent cardiovascular events in these patients^11^.

The Northern Sweden MONICA study showed that the presence of IGT in women is associated with a significantly higher incidence of painless myocardial ischemia^12^. Michal Mazurek et al. investigated the relationship between newly diagnosed glucose metabolism disorders in patients who had just undergone MI, and the frequency of deaths during a prospective follow-up period of an average of 3 years. It was shown that DM and IGT have an equally pronounced negative prognostic effect on the frequency of deaths, while IFG does not^13^.

Experimental studies evaluating the effect of IGT on functional and morphological changes in the myocardium are rare. Jia-Liang Liang et al. in 2011 showed that rats with IGT have a decrease in myocardial ejection fraction, as well as an increase in the number of cardiomyocytes prone to apoptosis in the absence of acute myocardial ischemia modeling^14^.

We have not found any experimental work in which the myocardial necrosis volume was investigated under IGT conditions. At the same time, these are the experimental conditions that make it is possible to create a "pure model" of MI and IGT, which in turn allows to study the direct effect of IGT on both hemodynamic parameters and myocardial necrosis volume, as well as to study the glucose-lowering drugs cardioprotective potential.

The objectives of our study were:

- to compare the severity of myocardial damage caused by transient ischemia ex vivo in animals with IGT, experimental type 2 diabetes and in animals without disorders of glucose metabolism.
- to study the potential cardioprotective effect of metformin (MET) and a glucagon-like peptide-1 (GLP-1) receptor agonist liraglutide (LIRA) in animals with IGT and to compare the prominence of this effect in transient myocardial ischemia ex vivo.

We chose MET as it is the first-line drug for DM therapy^15^, which is often used for IGT and has shown its effectiveness in patients with IGT in terms of preventing the development of DM. For GLP-1 receptor agonists, numerous pleotropic effects have been described in recent years, among which the cardioprotective effect occupies one of the leading positions. Thus, LIRA therapy, according to the LEADER study, demonstrated the ability to reduce overall mortality, mainly by reducing cardiovascular mortality^16^. Currently, GLP-1 receptor agonists are recommended for both primary and secondary prevention of cardio-vascular diseases.

## Methods

### Animals

The study was carried out in male Wistar rats weighing 150–250 g (n = 32).

The animals were maintained in fixed light mode, 12.00: 12.00 h (light: dark), no more than 5 animals per cage with free access to food and water. The temperature was maintained within the range of 22–25 °C, the relative humidity—50–70%.

The duration of quarantine (acclimatization period) for all animals was 14 days. During the quarantine, every animal was examined daily. The color of the skin and visible mucous membranes, behavior, the motor activity, the presence of seizures, changes in the respiratory movements, and tail position were assessed. Weighing was carried out upon arrival of the animals and during the quarantine period—at least once a week. Animals with deviations in weight, general condition or behavior were not included in the experiment.

### Induction of type 2 diabetes mellitus and impaired glucose tolerance

Animals were kept on the diet with increased amount of saturated fat (BioPro, Novosibirsk, Russian Federation: exchange energy 2690 kcal/kg, crude protein 20%, crude fat 22%)^17^ (further: high-fat diet, HFD) during all the experiment.

After first 4 weeks of HFD a solution of nicotinamide (Nicotinamide, Sigma-Aldrich, St. Louis, MO, USA) 230 mg/kg was injected intraperitoneally as a pancreatic protector, after 15 min—a solution of streptozotocin (Streptozocin, Sigma-Aldrich, St. Louis, MO, USA) 60 mg/kg intraperitoneally as a pancreatic toxin^18^.

On the second and third days after the administration of nicotinamide and streptozotocin, glycemia was determined. For this, a tail vein puncture was performed, after which the glucose content in the obtained venous blood drop was determined using an Accu-Chek Performa glucometer (Roshe, Germany). Glycemic values from 3.3 to 7.8 mmol/L were considered normal, since the measurement was made during the day (not fasting). DM was diagnosed when two measurements performed in different days showed glycemia elevation more than 11.1 mmol/L^19,20^. If lower glycemic values ​​were found in at least one of the measurements, an oral glucose tolerance test (OGTT) was performed. Glycemia was measured initially (fasting), as well as 15, 30 and 60 min after the gastric administration of a 40% glucose solution 3 g/kg of animal body weight. If we detected glycemia more than 11.1 mmol/L at any of the measurement points during OGTT we diagnosed the presence of DM. If glycemia was in the interval of 7.8–11.0 mmol/L we diagnosed IGT^21^. Animals whose glycemic parameters at all measurement points did not exceed 7.8 mmol/L were regarded as having no disturbances in glucose metabolism and were excluded from further experiment.

Glycemic measurements for making the diagnosis of DM and IGT are shown in Table 1.

**Table 1:** Blood glucose measurement for DM and IGT diagnostics.

Group	Animal №	Blood glucose level, mmol/L						
		1st measurement	2nd measurement	ORAL GLUCOSE TOLERANCE TEST				
				Baseline	15 min	30 min	60 min	90 min
DM	1	16.0	12.1					
	2	20.5	21.6					
	3	19.2	16.0					
	4	9.7	8.6	3.8	10.5	17.6	18.0	15.3
IGT	5	9.9	7.7	4.2	7.0	9.3	10.2	10.9
	6	6.5	6.4	4.6	8.9	8.7	10.2	9.2
	7	6.1	6.3	3.8	5.7	7.1	9.5	8.0
	8	7.7	7.0	5.3	5.9	7.9	8.8	9.6
IGT + MET	9	6.8	5.7	4.2	10.1	10.4	9.3	9.9
	10	6.7	6.9	4.5	9.6	10.4	10.6	9.9
	11	5.6	5.8	5.4	7.7	9.2	9.7	9.2
	12	6.7	5.3	5.3	6.6	9.8	10.7	10.8
IGT + LIRA	13	8.6	8.5	4.6	6.9	8.1	7.3	9.0
	14	5.7	6.3	4.7	8.2	8.6	9.5	9.0
	15	5.4	8.0	4.5	7.4	7.7	9.8	8.5
	16	5.3	7.7	4.8	7.3	8.8	10.5	9.6

### Study groups

After the acclimatization period, the following experimental groups were formed:

1. «CRL» (n = 6)—control group—rats were fed with standard chow for 16 weeks.
2. «HFD» (n = 8)—high-fat diet group—rats were fed with high-fat diet for 16 weeks.
3. «DM» (n = 4)—type 2 diabetes mellitus group—after the induction of DM rats were kept without treatment for 12 weeks.
4. «IGT» (n = 4)—impaired glucose tolerance group—after the induction of IGT rats were kept without treatment for 12 weeks.
5. «IGT + MET» (n = 4)—impaired glucose tolerance + metformin group—4 weeks after the induction of IGT 8-weeks metformin therapy started.
6. «IGT + LIRA» (n = 4)—impaired glucose tolerance + liraglutide group—4 weeks after the induction of IGT 8-weeks liraglutide therapy started.

**Figure 1:**
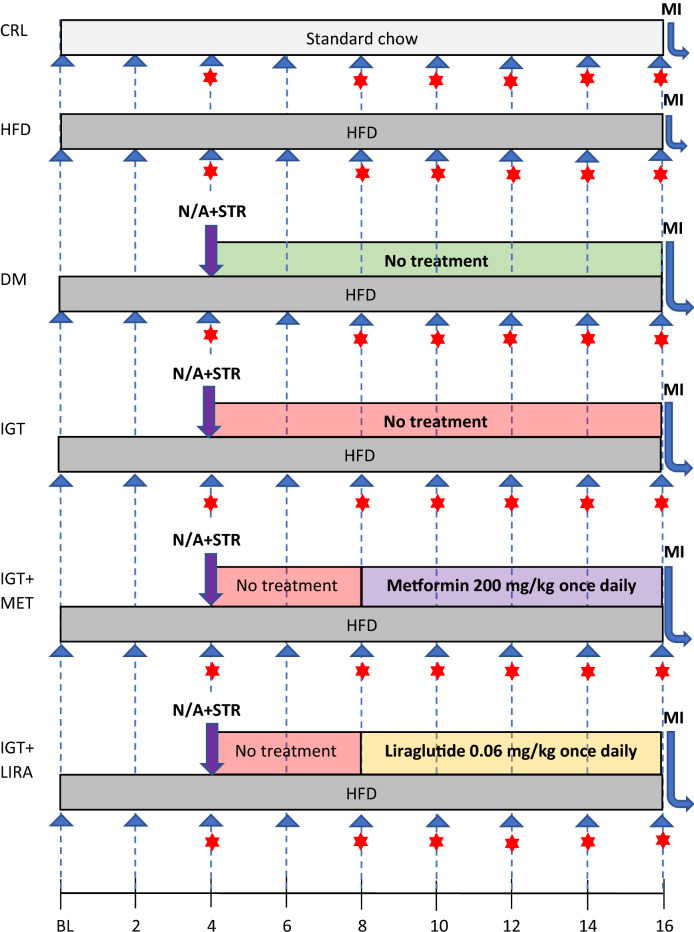
Study design. CRL (n = 6), control group; HFD (n = 8), high-fat diet group; DM (n = 4), type 2 diabetes mellitus group; IGT (n = 4), impaired glucose tolerance group; IGT + MET (n = 4), impaired glucose tolerance + metformin group; IGT + LIRA (n = 4), impaired glucose tolerance + liraglutide group; N/A, nicotinamide; STR, streptozotocin; MI, myocardial infarction modelling; BL, baseline; blue filled triangle, body weight and food consumption measurement; red filled star, blood glucose measurement.

The number of animals in these groups was determined during the experiment, depending on which variant of the carbohydrate metabolism disorder had developed after streptozotocin and nicotinamide injection (Fig. 1).

### Study drugs

Metformin powder (Metformin hydrochloride Sigma-Aldrich, St. Louis, MO, USA) was dissolved in distilled water and given per os by gastric tube 200 mg/kg of body weight once daily for 8 weeks (56 days).

Liraglutide (Victoza, NovoNordisk, Denmark) was administered subcutaneously 0.06 mg/kg of body weight once daily for 8 weeks (56 days).

### Body weight and food consumption measurement

Once every two days during the entire experiment, the animals were weighed, and the weight of the chow consumed in 2 days was determined.

### Glucose measurement

Glucose measurement was performed with the help of Accu-Chek Performa glucometer (Roshe, Germany).

In CRL and HFD groups glucose assessment was performed at the end of the 4th week (28th day), 8th week (56th day), 10th week (70th day), 12th week (84th day), 14th week (98th day) and 16th week (112th day) of experiment at the same daytime (not fasting measurement).

In DM and IGT groups glucose assessment was performed after 4 weeks of experiment on the 2nd and 3rd day after streptozotocin injection, at the end of the 8th week (56th day), 10th week (70th day), 12th week (84th day), 14th week (98th day) and 16th week (112th day) of experiment at the same daytime (not fasting measurement).

In IGT + MET and IGT + LIRA groups glucose assessment was performed after 4 weeks of experiment on the 2nd and 3rd day after streptozotocin injection, at the end of the 8th week (56th day), then every third day during the 8 weeks of treatment at the same daytime (not fasting measurement), 5 h after the certain treatment.

### Isolated heart perfusion according to Langendorff

Anesthesia was performed by Zoletil (tiletamine hydrochloride 30 mg/kg and zolazepam hydrochloride 30 mg/kg) intramuscularly and xylazine hydrochloride 6 mg/kg intramuscularly. After reaching the surgical stage of anesthesia (Zoletil + Xylazine IM), a wide thoracolaparotomy was performed, the organs of the chest cavity were exposed and the heart was removed, after which it was connected to the modified Langendorff apparatus. Perfusion was performed retrogradely through the ascending aorta, while venous outflow of perfusate occurred from the right chambers of the heart. A polyethylene balloon was inserted into the left ventricular cavity, connected to a pressure transducer to register intra-left ventricular pressure and create an adequate preload. Coronary perfusion volumetric rate was also recorded by measuring venous outflow. Perfusion was performed with modified Krebs–Henseleit buffer solution (consisting of the following [in mmol/L]: NaCl, 118.5; KCl, 4.7; NaHCO3, 25.0; KH2PO4, 1.2; MgSO4, 1.2; glucose, 11.0; and CaCl2, 1.5) at a constant pressure of 80 mm Hg and temperature + 37 °C^22^. In this case, the left ventricle contracted in isovolumetric mode due to the fact that the volume of the balloon introduced into its cavity was constant and provided preload at the physiological level (no more than 12 mm Hg). Intra-ventricular pressure was recorded using PhysExp software (Cardioprotect Ltd., Saint Petersburg, Russian Federation).

After the end of the stabilization period lasting 5 min, the functional parameters of the heart were recorded. Left ventricular systolic pressure (LVSP) and left ventricular end-diastolic pressure (LVEDP) were measured isovolumetrically using a nonelastic polyethylene balloon introduced into the left ventricle via the left atrium. Left ventricular developed pressure (LVDP) was calculated as the difference between LVSP and LVEDP. Intensity of coronary perfusion (coronary flow rate (CFR)) was determined by measuring the time for the collection of perfusate outflow.

Global 30-min normothermic myocardial ischemia and subsequent 90-min reperfusion were induced by reversible shutdown of perfusion. During the period of ischemia, the value of intra-left ventricular pressure was recorded every 5 min in order to assess the severity of ischemic contracture. During the reperfusion period, functional parameters (coronary blood flow and intra-ventricular pressure) were recorded every 15 min.

### Infarction size measurement

At the end of reperfusion, the volume of the irreversibly damaged myocardium was measured using the method of histochemical staining of transverse heart sections with 1% triphenyltetrazolium chloride solution. The sections were incubated in the indicated solution for 15 min and the viable myocardium was stained bright red. Areas of irreversibly damaged myocardium remained unstained. Then the sections photographs were made with a stereomicroscope (SMZ18; Nikon, Tokyo, Japan) coupled with a digital camera (DS-Fi2, Nikon, Tokyo, Japan) and computer processing of the images was performed. Infarction size was expressed as a percentage from total ventricular area minus the cavities.

### Statistical analysis

Statistical data processing was performed using the software package IBM SPSS Statistics-22 (IBM, USA) and Statistica-10 (Statsoft, USA).

The significance of differences between groups was assessed using the nonparametric Kruskal-Wallace and Mann–Whitney test for independent samples, using a nonparametric analysis of variance (a posteriori pairwise comparison of groups using the Dunn test). The significance of differences within one group was assessed using the nonparametric Friedman and Wilcoxon tests for dependent variables with the introduction of the Bonferroni correction with false discovery rate. All indicators are presented as "median (25%; 75%)". *P* values ​​less than 0.05 were considered significant.

### Ethics approval

All experimental procedures were performed in accordance with the Guide for the Care and Use of Laboratory Animals (NIH publication No. 85–23, revised 1996) and the European Convention for the Protection of Vertebrate Animals used for Experimental and other Scientific Purposes. The study protocol was approved by Institutional Animal Care and Use Committee of Almazov National Medical Research Centre (Protocol Number 19-1П3#V1, Jan 14 2019). All efforts were performed to protect the laboratory animals and minimize their suffering throughout the study. The experiments complied with the ARRIVE guidelines (http://www.nc3rs.org/ARRIVE).

## Results

Streptozotocin + nicotinamide administration failed to induce any impairment of glucose metabolism in 2 rats, that were therefore excluded from the further experiment. In 4 rats type 2 DM was observed according to the above mentioned criteria, 12 rats developed IGT, therefore the latter were divided into 3 groups 4 animals each (“IGT”, “IGT + MET”, “IGT + LIRA”).

### Body weight and food consumption

**Figure 2:**
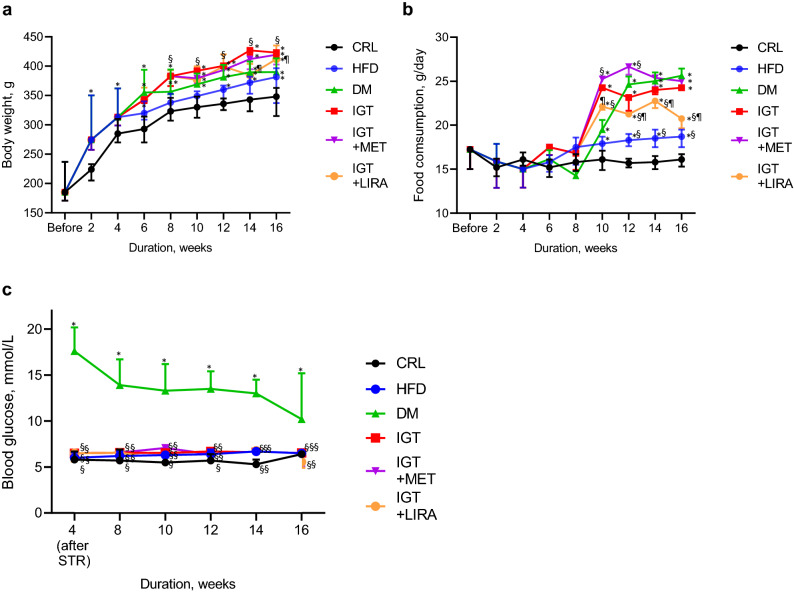
Dynamics in body weight, food consumption and glycemia during the experiment. (**a**) body weight, (**b**) food consumption, (**c**) blood glucose dynamics. Results are presented as median (25; 75) %. CRL (n = 6), control group; HFD (n = 8), high-fat diet group; DM (n = 4), type 2 diabetes mellitus group; IGT (n = 4), impaired glucose tolerance group; IGT + MET (n = 4), impaired glucose tolerance + metformin group; IGT + LIRA (n = 4), impaired glucose tolerance + liraglutide group. *p < 0.05, comparing with CRL group. §*p* < 0.05, comparing with DM group. ¶< 0.05, while comparing between groups IGT + MET and IGT + LIRA. STR, streptozotocin.

During the first 4 weeks of experiment rats receiving high-fat diet had significantly more intensive weight gain that those fed with standard chow (*p* < 0.05) (Fig. 2a, Table S1 online). After dividing into the study groups, it was primarily observed that weight gain in DM, IGT and HFD groups did not differ significantly among each other (*p* > 0.05) and was less prominent than in CRL group (*p* < 0.05). But after 8 weeks of experiment rats in IGT group demonstrated more intensive weight gain than rats in DM and HFD groups (*p* < 0.05 for each). Interestingly, animals with IGT treated with MET had similar weight gain as those with IGT without treatment (*p* > 0.05). On the other hand, treatment of IGT rats with LIRA slowed physiological body weight increase (Fig. 2a, Table S1 online), which reflects the known anorexigenic effect of LIRA^15^.

Food consumption in IGT group was similar to that in DM group and significantly exceeded food consumption in both CRL and HFD groups. HFD animals had slightly larger food consumption than CRL ones. MET administration to IGT animals in general did not decrease food intake comparing with IGT animals without therapy. On the other hand, treatment of IGT rats with LIRA led to significant decrease of chow consumption which was most prominent by the 16th week of experiment (Fig. 2b, Table S2 online).

### Blood glucose level

Animals in DM group had higher blood glucose level than animals in CRL, HFD, IGT, IGT + MET and IGT + LIRA groups. Of note, glycemic profile in IGT rats without treatment did not significantly differ from that in the rats with IGT treated with MET or LIRA. Similarly, MET and LIRA administration to IGT rats had comparable glucose-lowering effect, which, in our opinion, might be due to the fact that pre-treatment glucose level almost did not exceed normal range. Glycemia dynamics is shown in Fig. 2c and in Table S3 online. No hypoglycemic episodes were observed in any of the study groups.

### Isolated heart function and myocardial infarct size

**Figure 3:**
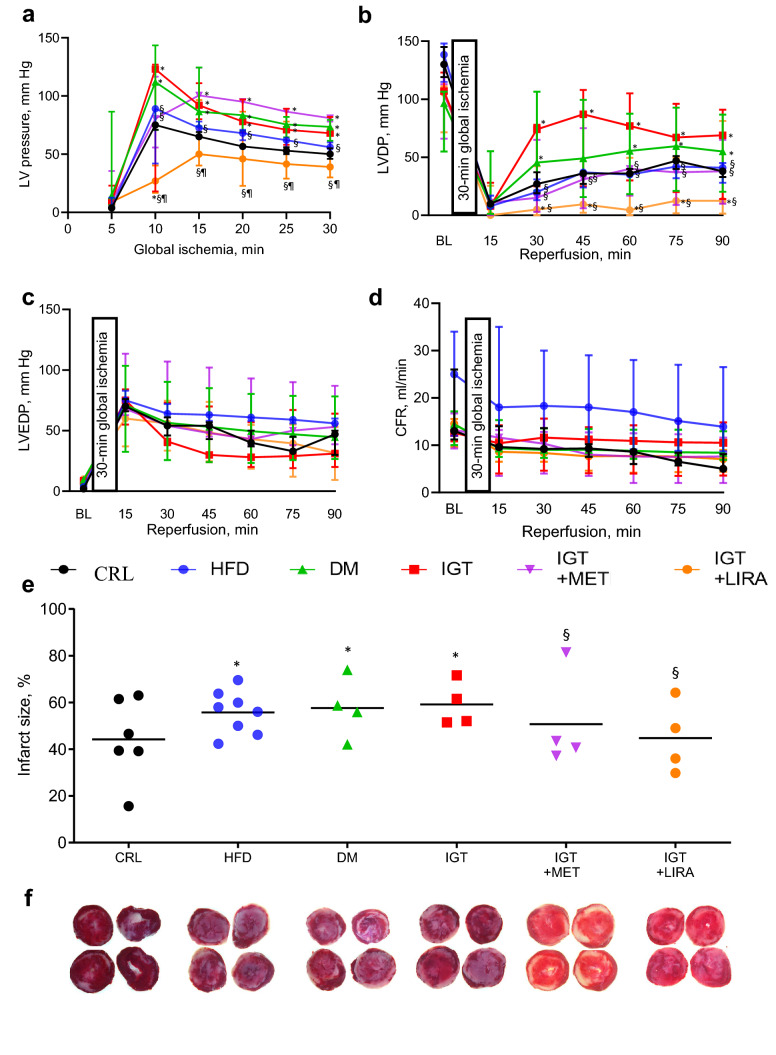
Functional parameters and myocardial infarct size in isolated rat hearts subjected to 30 min of global ischemia followed by 90 min of reperfusion. (**a**) Ischemic contracture, (**b**) LVDP, (**c**) LVEDP, (**d**) CFR values at baseline and during the experiment. Results are presented as median (25; 75) %. (**e**) Infarct size results are presented as dot plots with median values. (**f**) Representative images of heart slices stained with triphenyltetrazolium chloride. CRL (n = 6), control group; HFD (n = 8), high-fat diet group; DM (n = 4), type 2 diabetes mellitus group; IGT (n = 4), impaired glucose tolerance group; IGT + MET (n = 4), impaired glucose tolerance + metformin group; IGT + LIRA (n = 4), impaired glucose tolerance + liraglutide group. *p < 0.05, comparing with CRL group. §*p* < 0.05, comparing with DM group. ¶< 0.05, while comparing between groups IGT + MET and IGT + LIRA.

Changes in LVP that occurred during the global ischemia are shown in Fig. 3a and Table S4 online. Ischemic contracture was determined as follows: at least a threefold increase in LVP at any time during the ischemic period in comparison with the LVP after five minutes of ischemia. We observed the most prominent increase in left ventricle (LV) pressure in DM and IGT groups after 10 min of ischemia, no differences were found between these groups. LV pressure in IGT + MET group was similar to that in CRL and HFD groups and lower than that in DM and IGT groups in 10 min, but from the 15th min up to the end of ischemic period LV pressure in IGT + MET group caught up with that in IGT and DM groups. Of note, LV pressure in IGT + LIRA group was significantly lower than in IGT, DM groups and in IGT + MET group during all the ischemic period and did not differ from that in both CRL and HFD groups from 15 to 30 min, being even lower at the 10th minute (Fig. 3a, Table S4 online). Thus, we can conclude that LIRA administration in IGT effectively diminishes ischemic contracture.

The baseline LVDP values were similar in all the groups. LVDP in IGT group tended to be higher than in DM group for 30–60 min of reperfusion period, although the differences were not significant (*p* > 0.05). Interestingly, LVDP in IGT group was higher than in both CRL and HFD groups. Moreover, LVDP was lower in both IGT + MET and IGT + LIRA groups comparing with IGT and DM groups. No significant differences were observed between IGT + MET and IGT + LIRA groups, although LVDP tended to be lower in IGT + LIRA group, most probably due to lower LVSP (Fig. 3b, Table S5 online).

There were no significant differences in LVEDP (Fig. 3c, Table S6 online). Similarly, CFR did not differ among all the groups during reperfusion period, except HFD group, where CFR tended to be higher in 30–45 min of reperfusion (*p* > 0.05), comparing with all the other groups (Fig. 3d, Table S7 online).

Myocardial infarction size in DM group was larger than in CRL group (57.26 (45.51; 70.08) % and 42.98 (33.26; 61.84) %, respectively, *p* < 0.05). Of note, myocardial damage size was similarly large in IGT (56.76 (51.58; 69.07) %), HFD (56.98 (47.11; 62.83) %) and DM groups (57.26 (45.51; 70.08) %), *p* > 0.05 for multiple comparison. On the other hand, in both IGT + MET (42.11 (38.08; 71.96) %) and IGT + LIRA (42.50 (31.37; 60.40) %) groups myocardial infarction size was significantly smaller than in IGT or DM group (*p* < 0.05). No difference was found between IGT + MET and IGT + LIRA groups (*p* > 0.05). Importantly, myocardial damage size in IGT + MET and in IGT + LIRA groups was similar to that in CRL group of animals without any glucose metabolism impairments (*p* > 0.05) (Fig. 3e, Table S8 online). Representative images of heart slices stained with triphenyltetrazolium chloride are shown in Fig. 3f.

## Discussion

We have demonstrated that both type 2 DM and IGT have a negative effect on hemodynamic parameters and lead to the formation of pronounced structural changes in the myocardium in conditions of transient ischemia ex vivo. It is noteworthy that all hemodynamic parameters, during both ischemia and reperfusion period, coronary blood flow, as well as myocardial damage volume did not differ in animals with DM and IGT, although glycemia parameters in diabetic animals were significantly higher. This allows us to suggest that impairment of glucose metabolism, regardless of the severity of hyperglycemia, has a negative effect on the resistance of the myocardium to ischemic-reperfusion injury. Moreover, feeding with high-fat diet, resulting in obesity development, without glucose metabolism impairment, has similar negative impact on structural changes of the myocardium, as myocardial infarction volume in HFD animals did not differ from that in IGT and DM rats.

Both MET and LIRA have infarct-limiting properties in transient myocardial ischemia in rats with IGT. We did not observe any significant difference in the prominence of infarct-limiting action of these two drugs. The myocardial infarction volume in IGT animals treated with both MET and LIRA was significantly smaller than that in type 2 diabetic animals and in animals with IGT without treatment.

At the same time, only LIRA, but not MET, has a positive influence on hemodynamic parameters, predominantly decreasing ischemic contracture, which might comprise one of mechanisms of LIRA cardioprotective action.

As to our knowledge, our study was the first that evaluated and compared cardioprotective properties of MET and LIRA in rats with IGT. There are a few data elucidating cardioprotective potential of MET in the clinical conditions, in patients with prediabetes and even in individuals without impairment of glucose metabolism^23–26^. Some of these trials predominantly focus on the evaluation of surrogate markers potentially characterizing cardiovascular risk. Thus, Chris P.H. Lexis et al. investigated the effects of 4-months MET therapy in patients without glucose metabolism abnormalities having undergone MI on cardiovascular risk factors and revealed that MET causes improvement of glycated hemoglobin, total cholesterol, low-density cholesterol, body mass index, comparing with placebo^23^.

It has also been demonstrated that patients with stable angina and IGT receiving MET therapy have better prognosis than individuals with stable angina and IGT without treatment which is characterized by lower incidence of major adverse cardiac events in the follow-up observation period^24^.

There are some data about cardioprotective properties of MET in animals without glucose metabolism impairments. For example, Hamid Soraya et al. showed that administration of MET for the short period of time while modelling isoproterenol-induced MI in healthy rats causes significant infarct-limiting effect^27^. Nevertheless, we have not found experimental works evaluating infarct-limiting effect of MET in prediabetic conditions. Therefore, we may assume that the present study is the first elucidating its cardioprotective potential in transient global myocardial ischemia.

Of note, MET influence on hemodynamic parameters, both in clinical and experimental trails, are contradictory. Thus, diastolic function measured by tissue Doppler imaging in patients with DM was shown to be improved by MET treatment after coronary angiography^28^. Left ventricular end-diastolic pressure was lowered in a non-diabetic rat model of post-MI heart failure^25^. However, in conditions of ST-elevation MI in patients without impairment of glucose metabolism, MET failed to preserve left ventricular ejection fraction at 4 months of treatment^23^. In the meta-analysis including 4 randomized clinical trials involving 1366 non-diabetic patients MET was found to significantly reduce left ventricular ejection fraction and increase both left ventricular end-diastolic and left-systolic volume^26^. These data correlate with the results of our study, where we observed significant infarct-limiting effect of MET although no positive influence on hemodynamic parameters was discovered, including no influence on ischemic contracture and no certain improvement of reperfusion hemodynamics.

Interestingly, it is thought that potential cardioprotective effect of MET might be due to endothelial dysfunction diminishing. Thus, it has been shown that patients with IGT receiving MET therapy have less prominent endothelial dysfunction of left anterior descending coronary artery evaluated by acetylcholine probe during coronary angiography than those with IGT without MET treatment^24^. Earlier we have also investigated endothelial protective effect of MET in comparison with LIRA in patients with type 2 DM^29^. We demonstrated that 9-months treatment with MET improves vasomotor endothelial function characterized by decrease of circulating endothelin-1 level, although this action was strongly connected with glycemic profile normalization and was diminished in patients with glycated hemoglobin increase. Moreover, we did not observe any positive effect of MET on endothelial-dependent vasodilation studied by acetylcholine electrophoresis. Taken together, these data imply that MET does not have its own endothelial-protective property, without connection with its influence on glucose metabolism, in diabetic conditions, although it might have an endothelial protective potential in prediabetes. On the other hand, even in prediabetic conditions we cannot completely exclude the potential connection of MET endothelial protective effect with better glucose profile under the use of this drug.

According to the literature data, effects of MET are realized by means of a direct inhibition of complex 1 of the respiratory chain in mitochondria, which has been demonstrated for its anti-diabetic action^30^ and might form the basis for cardioprotective effect. Cardioprotective property of MET is thought to be connected with adenosine monophosphate activated protein kinase (AMPK) activation. AMPK stimulates the energy conserving metabolic processes which leads to increased tolerance to any hypoxia, including that taking place during ischemia–reperfusion injury^31^. Regarding myocardium, the AMPK system is prominently activated by hypoxia only under pathological ischemic circumstances, whereas AMPK-activating drugs like MET mimic these effects of hypoxia. The AMPK activation by means of MET causes glucose uptake increase and stimulates glycolysis in the cardiomyocytes and thereby makes the heart more resistant in the future ischemic conditions, serving the aims of pharmacological preconditioning^32^. AMPK activation by MET reverses AMPK inactivation that happens in oxidative stress conditions and prevents mitochondrial DNA damage^33^. AMPK activation also suppresses cell growth and proliferation and thereby prevents myocardial remodeling after MI^27^, which might be important in the conditions of chronic experiment and could not be realized in our study.

Besides, MET abolishes oxidative stress-induced interactions between peroxisome proliferator-activated receptor alpha and cyclophilin D, while the annihilation of these interactions is associated with permeability transition pore opening inhibition^33,34^. Notably, this process is independent on phosphorylation and acetylation of peroxisome proliferator-activated receptor alpha and cyclophilin D^33^.

Of note, no clinical trials have been found evaluating the opportunity of LIRA to decrease the incidence and reduce severity of MI or to improve the stable angina development in IGT conditions, though its cardioprotective effect in type 2 DM is well known^16,35,36^. It has only been demonstrated that liraglutide 3.0 mg, approved for the treatment of obese patients without DM, reduces incidence of DM development in obese patients with prediabetes. In the SCALE Obesity and Prediabetes study, in IGT patients LIRA 3.0 mg therapy showed high effectiveness in body mass index reduction which might be interpreted as decrease of cardiovascular risk factor^37^. Most probable reason for lack of abovementioned data is that LIRA has not been approved for the use in IGT which limits its practical use and therefore its study in this pathology.

Similarly to the situation observed for MET, no experimental studies elucidating cardioprotective effect of LIRA in myocardial ischemic-reperfusion injury in animals with prediabetes have been found. Therefore, our study seems to be the first one describing the influence of LIRA on hemodynamic parameters and myocardial damage volume in experimental conditions of IGT.

On the other hand, there is a rather big number of investigations aimed to determine the cardioprotective effect of LIRA in both animals without glucose metabolism abnormalities and with DM.

Thus, the use of LIRA in animals without DM increased the survival rate after MI, decreased the necrosis volume and the severity of cardiac hypertrophy manifestations. Similarly, treatment with LIRA in mice with experimental type 2 DM increased survival compared with placebo, which was not observed with MET, despite similarly satisfactory glycemic control^38^.

D.-D. Huang et al. demonstrated that the administration of LIRA to rats without DM after experimental MI improves cardiac function and slows down the fibrosis development^39^. Potential mechanism of LIRA cardioprotective action both in normal glycemic status and in DM is supposed to be connected with endothelial dysfunction diminishing. Thus, it has been shown that LIRA increases NO production, decreases edothelin-1 level, normalizing vasomotor endothelial function, and increases microvasculature blood flow in obese rats^40^. Similarly, the use of LIRA increases nitric oxide level in diabetic rats^41^.

Also, it has been described that in DM LIRA protective mechanisms might be due to positive influence on the manifestations of insulin resistance and adiposopathy which is characterized by adiponectin increase. Similar mechanisms might also have an impact it IGT^41^.

Interestingly, some of the cardioprotective effects of GLP-1 receptor agonists administration during ischemia/reperfusion injury are preserved in isolated hearts of GLP-1 receptor knock-out mice, suggesting the existence of a GLP-1 receptor independent pathway of cardioprotection. At the same time, administration of GLP-1(9–36), product of GLP-1 degradation by dipeptidylpeptidase-4, to GLP-1 receptor knock-out animals results in ischemic damage reduction, suggesting that GLP-1(9–36) might be an activator of the GLP-1 receptor independent pathway associated with cardioprotection^42^.

## Conclusions

Thus, we can conclude that IGT and DM have similarly pronounced negative influence on the resistance of myocardium to ischemic-reperfusion injury, regardless hyperglycemia severity. Obesity development also has negative impact on myocardial structural damage. Both MET and LIRA have cardioprotective effect that is independent on their influence on glucose metabolism. Cardioprotective property of LIRA includes both improvement of hemodynamic parameters and infarct-limiting action, whereas use of MET only decreases myocardial damage volume. We can suppose that cardioprotective action of LIRA is more prominent in transient myocardial ischemia in IGT rats. The received data might open the perspectives for clinical investigation of potential cardioprotective effects of both MET and LIRA in patients with IGT.

### Study limitations

The limitations of this study might be connected with small number of experimental animals included, as we aimed to phenomenologically reveal the presence or absence of any differences in myocardial damage parameters in diabetic and IGT conditions, as well as MET and LIRA influence on these parameters. Further investigations are required involving larger number of experimental animals, including the study of the underlying mechanisms. Of note, we observed statistically significant differences between study groups, which seems to make the results reliable.

## Supplementary Information

Supplementary Tables.

## Data Availability

The data generated and analyzed during this study are included in this published article and its supplementary information files. Additional information is available from the corresponding author on reasonable request.

## Abbreviations

IGT: Impaired glucose tolerance
MI: Myocardial infarction
DM: Diabetes mellitus
MET: Metformin
LIRA: Liraglutide
IDF: International Diabetes Federation
IFG: Impaired fasting glucose
GAMI: “Glucose Tolerance in Patients with acute myocardial infarction” [study]
ECG: Electrocardiogram
GLP-1: Glucagon-like peptide-1
OGTT: Oral glucose tolerance test
HFD: High-fat diet
N/A: Nicotinamide
STR: Streptozotocin
CRL: Control [group]
BL: Baseline
LVSP: Left ventricular systolic pressure
LVEDP: Left ventricular end-diastolic pressure
LVDP: Left ventricular developed pressure
CFR: Coronary flow rate
LV: Left ventricle
AMPK: Adenosine monophosphate activated protein kinase
DNA: Deoxyribonucleic acid
