# Supplementary material for: Comparative evaluation of metformin and liraglutide cardioprotective effect in rats with impaired glucose tolerance
Source: Sci Rep. 2021 Mar 23;11:6700. doi: 10.1038/s41598-021-86132-2 (PMC7987997; doi:10.1038/s41598-021-86132-2)
Supplement: Supplementary file 1 — Supplementary Tables. [file 41598_2021_86132_MOESM1_ESM.pdf]

Comparative evaluation of metformin and liraglutide cardioprotective effect in rats with impaired glucose tolerance

Anna Simanenkova, Sarkis Minasian, Tatiana Karonova, Timur Vlasov, Natalya Timkina, Oksana Shpilevaya, Aleksandra Khalzova, Anzhelika Shimshilashvili, Valeria Timofeeva, Daniil Samsonov, Yury Borshchev, Michael Galagudza

Supplementary Table S1. Body weight measurement during the study.

Time of measurement	Body weight, g					
	**CRL**	**HFD**	**DM**	**IGT**	**IGT+MET**	**IGT+LIRA**
**Before**	185.00 (170.75; 236.75)	185.00 (170.75; 236.75)	185.00 (170.75; 236.75)	185.00 (170.75; 236.75)	185.00 (170.75; 236.75)	185.00 (170.75; 236.75)
**2 weeks**	224.00 (205.00; 233.00)	274.50 (257.25; 350.25)	274.50 (257.25; 350.25)	274.50 (257.25; 350.25)	274.50 (257.25; 350.25)	274.50 (257.25; 350.25)
**4 weeks**	285.00 (270.00; 310.00)	313.00 (298.75; 362.00)	313.00 (298.75; 362.00)	313.00 (298.75; 362.00)	313.00 (298.75; 362.00)	313.00 (298.75; 362.00)
**6 weeks**	293.00 (270.00; 310.00)	320.00 (309.00; 340.00)	355.00 (319.50; 393.88)	343.00 (337.00; 363.50)	343.00 (337.00; 363.50)	343.00 (337.00; 363.50)
**8 weeks**	323.00 (307.00; 346.00)	338.00 (325.00; 353.00)	356.50 (322.50; 394.25)	383.00 (366.00; 393.00)	383.00 (366.00; 393.00)	383.00 (366.00; 393.00)
**10 weeks**	330.00 (312.00; 348.00)	349.00 (329.00; 357.00)	369.00 (353.00; 387.50)	392.00 (391.50; 401.50)	379.50 (370.25; 398.25)	376.50 (367.50; 399.75)
**12 weeks**	336.00 (325.00; 345.00)	360.00 (337.00; 367.00)	381.25 (364.37; 402.25)	400.5 (398.75; 413.25)	393.25 (379.00; 418.50)	399.50 (394.75; 420.25)
**14 weeks**	343.00 (323.00; 348.00)	372.00 (353.00; 382.00)	389.50 (380.75; 408.00)	427.00 (419.50; 437.00)	412.00 (394.75; 439.50)	384.75 (381.62; 403.88)
**16 weeks**	348.00 (315.00; 363.00)	381.5 (337.00; 396.50)	390.00 (377.00; 413.50)	423.00 (416.00; 435.00)	419.50 (402.75; 445.25)	412.00 (406.75; 435.25)

Results are presented as median (25; 75) %.

Supplementary Table S2. Food consumption during the study.

Time of measurement	Food consumption, g/day					
	CRL	HFD	DM	IGT	IGT+MET	IGT+LIRA
**Before**	17.25 (15.00; 17.50)	17.25 (15.00; 17.50)	17.25 (15.00; 17.50)	17.25 (15.00; 17.50)	17.25 (15.00; 17.50)	17.25 (15.00; 17.50)
**2 weeks**	15.20 (14.20; 16.20)	15.88 (12.88; 17.88)	15.88 (12.88; 17.88)	15.88 (12.88; 17.88)	15.88 (12.88; 17.88)	15.88 (12.88; 17.88)
**4 weeks**	16.10 (15.40; 16.90)	15.00 (12.90; 16.00)	15.00 (12.90; 16.00)	15.00 (12.90; 16.00)	15.00 (12.90; 16.00)	15.00 (12.90; 16.00)
**6 weeks**	15.20 (14.10; 16.10)	15.80 (14.70; 16.60)	16.15 (16.05; 17.15)	17.50 (16.62; 18.75)	17.50 (16.62; 18.75)	17.50 (16.62; 18.75)
**8 weeks**	15.80 (14.80; 16.70)	17.50 (15.50; 18.60)	14.25 (13.50; 14.75)	16.81 (14.93; 17.75)	16.81 (14.93; 17.75)	16.81 (14.93; 17.75)
**10 weeks**	16.10 (14.90; 17.10)	17.90 (17.10; 18.70)	19.50 (18.50; 20.60)	24.25 (24.25; 24.50)	25.25 (25.25; 25.25)	22.13 (21.75; 22.5)
**12 weeks**	15.70 (15.20; 16.20)	18.30 (17.60; 19.00)	24.63 (22.63; 25.53)	23.15 (21.65; 24.15)	26.65 (25.74; 26.65)	21.25 (21.25; 21.25)
**14 weeks**	15.80 (15.00; 16.50)	18.50 (17.50; 19.50)	25.00 (23.00; 26.00)	24.00 (23.50; 24.35)	25.38 (24.38; 26.38)	22.75 (21.95; 23.75)
**16 weeks**	16.10 (15.30; 16.70)	18.70 (17.50; 19.50)	25.63 (24.93; 26.43)	24.25 (24.00; 25.75)	25.00 (24.00; 26.00)	20.75 (19.65; 21.55)

Results are presented as median (25; 75) %.

Supplementary Table S3. Blood glucose level during the study.

Time of measurement	Blood glucose, mmol/L					
	CRL	HFD	DM	IGT	IGT+MET	IGT+LIRA
**4 weeks (after streptozotocin)**	5.8 (5.5; 6.7)	6.0 (5.8; 6.6)	17.6 (11.3; 20.2)	6.5 (5.6; 6.8)	6.5 (5.6; 6.8)	6.5 (5.6; 6.8)
**8 weeks**	5.7 (5.6; 6.9)	6.2 (5.6; 6.7)	13.9 (12.4; 16.7)	6.6 (6.1; 7.1)	6.6 (6.1; 7.1)	6.6 (6.1; 7.1)
**10 weeks**	5.5 (5.2; 5.7)	6.3 (5.8; 6.5)	13.3 (12.0; 16.2)	6.5 (5.7; 8.3)	7.1 (6.7; 9.7)	5.8 (5.6; 6.9)
**12 weeks**	5.7 (5.5; 6.4)	6.4 (5.7; 6.9)	13.5 (12.7; 15.4)	6.7 (5.8; 7.8)	6.4 (6.0; 6.5)	5.4 (4.8; 6.2)
**14 weeks**	5.3 (4.9; 5.8)	6.7 (6.1; 6.9)	13.0 (12.3; 14.5)	6.6 (5.8; 7.5)	5.6 (5.4; 6.2)	6.8 (5.5; 8.0)
**16 weeks**	6.4 (5.8; 6.7)	6.5 (5.9; 6.8)	10.2 (7.3; 15.2)	6.5 (6.4; 6.6)	6.1 (5.1; 7.1)	5.6 (4.9; 7.2)

Results are presented as median (25; 75) %.

Supplementary Table S4. LV pressure during ischemia.

Time of measurement, min	LV pressure, mm Hg					
	CRL	HFD	DM	IGT	IGT+MET	IGT+LIRA
**5**	3.8 (1.6; 4.8)	4.0 (3.0; 12.7)	14.5 (6.3; 86.5)	9.0 (7.0; 23.0)	9.5 (6.8; 35.5)	9.0 (5.3; 9.8)
**10**	75.0 (71.0; 76.0)	89.0 (42.0; 91.0)	112.0 (70.8; 143.5)	123.0 (18.0; 127.0)	80.5 (55.8; 116.5)	27.0 (17.8; 40.3)
**15**	65.0 (64.0; 69.0)	72.6 (72.0; 78.0)	86.5 (74.0; 124.5)	92.0 (80.0; 111.0)	100.5 (65.5; 124.3)	50.0 (40.3; 96.5)
**20**	56.7 (56.0; 59.0)	68.0 (62.0; 71.0)	83.5 (70.3; 84.8)	78.0 (69.0; 97.0)	95.0 (92.3; 97.75)	46.0 (22.8; 86.5)
**25**	53.0 (50.0; 56.0)	62.0 (56.0; 64.0)	75.5 (64.5; 82.0)	71.0 (58.0; 89.0)	86.5 (83.8; 89.3)	41.5 (29.0; 78.0)
**30**	50.0 (46.0; 55.0)	56.0 (54.0; 60.0)	73.5 (61.5; 79.5)	68.0 (51.0; 82.0)	81.0 (78.0; 81.8)	39.0 (30.0; 72.0)

Results are presented as median (25; 75) %.

Supplementary Table S5. LVDP at baseline and during reperfusion.

Time of measurement, min	LVDP, mm Hg					
	CRL	HFD	DM	IGT	IGT+MET	IGT+LIRA
baseline	130.0 (119.0; 145.0)	138.4 (115.0; 148.0)	96.5 (55.0; 106.5)	107.0 (105.0; 123.0)	104.5 (66.0; 113.0)	105.5 (71.5; 111.0)
15	10.0 (8.0; 11.0)	8.0 (7.0; 17.0)	9.5 (1.3; 55.3)	8.0 (8.0; 28.0)	11.0 (0.0; 12.0)	0.0 (0.0; 25.5)
30	27.0 (19.0; 37.0)	20.0 (13.0; 31.0)	45.5 (21.3; 106.5)	74.0 (25.0; 78.0)	15.0 (3.0; 65.0)	5.0 (0.0; 42.25)
45	36.0 (27.0; 38.0)	37.0 (24.0; 37.0)	49.0 (15.8; 99.5)	87.0 (25.0; 108.0)	31.0 (6.0; 75.0)	9.5 (2.3; 46.8)
60	36.0 (35.0; 40.0)	35.0 (34.0; 36.0)	55.5 (18.3; 87.5)	77.0 (30.0; 105.0)	40.0 (17.0; 75.0)	4.5 (0.0; 49.5)
75	47.0 (40.0; 51.0)	42.0 (32.0; 44.0)	59.5 (21.0; 92.8)	67.0 (20.0; 96.0)	37.0 (9.0; 69.0)	12.5 (1.8; 49.5)
90	38.0 (33.0; 38.0)	41.0 (28.0; 45.0)	55.0 (20.3; 86.8)	69.0 (14.0; 91.0)	38.0 (10.0; 65.0)	12.5 (1.5; 81.3)

Results are presented as median (25; 75) %.

Supplementary Table S6. LVEDP at baseline and during reperfusion.

Time of measurement, min	LVEDP, mm Hg					
	CRL	HFD	DM	IGT	IGT+MET	IGT+LIRA
baseline	2.0 (2.0; 3.0)	3.6 (2.0; 4.0)	9.5 (5.3; 10.0)	8.0 (6.0; 11.0)	8.5 (4.75; 10.0)	10.0 (8.3; 11.8)
15	70.0 (66.0; 76.0)	75.0 (71.0; 83.0)	71.0 (32.5; 103.5)	75.0 (55.0; 84.0)	68.0 (62.75; 113.5)	60.0 (37.0; 66.5)
30	54.0 (46.0; 61.0)	64.0 (62.0; 72.0)	56.5 (25.8; 90.3)	41.0 (38.0; 73.0)	54.0 (52.0; 107.0)	56.0 (33.5; 74.8)
45	54.0 (43.0; 57.0)	63.0 958.0; 64.0)	53.0 (24.0; 85.0)	30.0 (24.0; 70.0)	48.0 (45.0; 102.0)	48.5 (24.8; 73.8)
60	40.0 (37.0; 50.0)	61.0 (58.0; 62.0)	49.5 (23.3; 80.3)	28.0 (20.0; 57.0)	43.0 (38.5; 93.0)	43.0 (18.5; 54.8)
75	33.0 (33.0; 45.0)	59.0 (56.0; 61.0)	47.0 (25.8; 78.3)	29.0 (19.0; 67.0)	50.0 (38.0; 90.0)	39.5 (12.0; 55.0)
90	47.0 (30.0; 50.0)	56.0 (55.0; 60.0)	44.5 (26.5; 78.3)	31.0 (20.0; 64.0)	53.0 (38.75; 87.0)	31.5 (9.25; 53.0)

Results are presented as median (25; 75) %.

Supplementary Table S7. CFR at baseline and during reperfusion.

Time of measurement, min	CFR, ml/min					
	CRL	HFD	DM	IGT	IGT+MET	IGT+LIRA
baseline	13.0 (11.2; 26.0)	25.0 (12.0; 34.0)	14.4 (10.0; 17.2)	12.6 (10.8; 12.7)	13.2 (9.3; 16.7)	14.6 (9.7; 15.6)
15	9.6 (9.2; 14.0)	18.0 (9.2; 35.0)	9.4 (7.5; 15.3)	10.4 (4.0; 14.2)	11.6 (3.5; 13.2)	8.6 (6.5; 11.5)
30	9.2 (9.0; 13.6)	18.3 (9.0; 30.0)	9.0 (7.3; 13.6)	11.6 (4.6; 15.6)	10.3 (4.0; 12.8)	8.4 (6.5; 11.1)
45	9.3 (7.5; 10.2)	18.0 (9.1; 29.0)	9.1 (7.6; 13.5)	11.2 (4.1; 13.8)	8.0 (3.5; 12.7)	7.7 (4.6; 10.7)
60	8.6 (6.0; 8.6)	17.0 (9.1; 28.0)	8.8 (7.1; 13.3)	10.9 (4.0; 14.2)	7.6 (2.0; 12.5)	7.8 (4.2; 10.7)
75	6.5 (5.7; 8.4)	15.1 (8.8; 27.0)	8.5 (6.8; 13.3)	10.6 (3.5; 15.1)	7.5 (2.0; 12.3)	7.6 (4.3; 10.3)
90	5.0 (5.0; 8.1)	13.9 (8.7; 26.5)	8.4 (6.9; 13.0)	10.5 (3.6; 14.8)	7.6 (2.0; 11.6)	7.1 (4.4; 9.0)

Results are presented as median (25; 75) %.

Supplementary Table S8. Infarct size.

	Group					
	CRL	HFD	DM	IGT	IGT+MET	IGT+LIRA
infarct size, %	42.98 (33.26; 61.84)	56.98 (47.11; 62.83)	57.26 (45.51; 70.08)	56.76 (51.58; 69.07)	42.11 (38.08; 71.96)	42.5 (31.37; 60.4)

Results are presented as median (25; 75) %.
